# Synthetic Colonic Mucus Enables the Development of Modular Microbiome Organoids

**DOI:** 10.21203/rs.3.rs-3164407/v1

**Published:** 2023-08-03

**Authors:** Scott Medina, Michael Miller

**Affiliations:** Pennsylvania State University; Pennsylvania State University

## Abstract

The human colon is home to more than a trillion microorganisms that modulate diverse gastrointestinal processes and pathophysiologies. Our understanding of how this gut ecosystem impacts human health, although evolving, is still in its nascent stages and has been slowed by the lack of accessible and scalable tools suitable to studying complex host-mucus-microbe interactions. In this work, we report a synthetic gel-like material capable of recapitulating the varied structural, mechanical, and biochemical profiles of native human colonic mucus to develop compositionally simple microbiome screening platforms with broad utility in microbiology and drug discovery. The viscous fibrillar material is realized through the templated assembly of a fluorine-rich amino acid at liquid-liquid phase separated interfaces. The fluorine-assisted mucus surrogate (FAMS) can be decorated with various mucins to serve as a habitat for microbial colonization and be integrated with human colorectal epithelial cells to generate multicellular artificial mucosae, which we refer to as a microbiome organoid. Notably, FAMS are made with inexpensive and commercially available materials, and can be generated using simple protocols and standard laboratory hardware. As a result, this platform can be broadly incorporated into various laboratory settings to advance our understanding of probiotic biology and inform *in vivo* approaches. If implemented into high throughput screening approaches, FAMS may represent a valuable tool in drug discovery to study compound metabolism and gut permeability, with an exemplary demonstration of this utility presented here.

The human gastrointestinal (GI) tract is host to a microbiome community of trillions of bacteria, diversified by around a thousand unique species.^1, 2^ These commensal microbes play critical roles in nutrient metabolism, immune training and prevention of opportunistic infections.^3, 4^ Imbalance in the GI bacterial consortium is now implicated in the pathophysiology of several human diseases, including inflammatory bowel disorders,^5^ type 2 diabetes,^6^ and certain cancers.^7^ The gut microbiome also impacts distal systems, including the brain where neurologic processes are altered via communication through the gut-brain axis.^8, 9^ Yet, despite its link to human health, our understanding of the functional properties of the gut microbiome, and how gut ecology influences human physiology, remains incomplete.

This gap in knowledge is largely due to a lack of facile and accessible tools suitable to studying, and controllably manipulating, complex host-mucus-microbe interactions.^10^ Although gnotobiotic mice remain the gold standard, these expensive models are inaccessible for many researchers and do not always faithfully replicate human disease. In addition, it remains difficult to carefully shape microbiome composition and control mucus composition in the *in vivo* environment.^11, 12^ Tractable *ex vivo* models of the GI microbiome therefore represent powerful tools to study host-microbiota interactions in a multiparametric fashion at the cellular and molecular level.

Towards this goal, the development of gastrointestinal organoids – *in vitro* models of human intestinal epithelium – have expanded opportunities to study disease and probe microbe-host interactions *ex vivo*.^13, 14^ While these are valuable additions to the modeling spectrum, three-dimensional systems are expensive, require significant technical training, and can be limited by access to primary material, making them difficult to implement in high throughput applications. Monolayer cultures can address these limitations but are currently unable to faithfully replicate the colonic mucus, which is composed of inner and outer mucus layers that possess varied structural morphologies and densities.

Here, we report a self-assembling gel that can be engineered to replicate the diverse mechanical, structural, and biochemical profiles of colonic mucus. The material is generated from the interfacial organization of a non-natural amino acid at fluorous-water interphases to form a viscous, double layer, colloid of fibrillar and coacervate assemblies. We demonstrate this synthetic platform can be readily coated with a variety of mucin proteins and directly incorporated into multicellular systems to create customizable *ex vivo* microbiome organoids. We show that the mechanical, structural, and biochemical properties can be independently tailored to create designer multicellular systems amenable to high throughput applications. As an exemplary demonstration, we incorporate these materials into a gastrointestinal permeability assay and demonstrate their ability to model oral bioavailability of macromolecular compounds.

## Fabrication and Molecular Characterization of Synthetic Mucus

**Figure 1:**
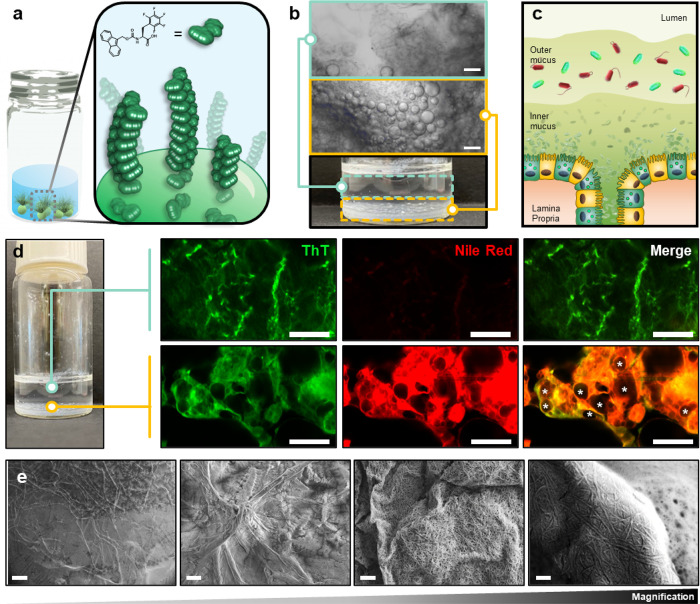
Development of double layer Fmoc-F_F_ fibrillar gels. **a**, Conceptual schematic of Fmoc-F_F_ assembly at fluorous-water interfaces to evolve percolating fibrils. **b**, Representative optical image and micrographs of Fmoc-F_F_ gels demonstrating the viscous PFD coacervate bottom layer (yellow) and fibrillar upper layer (teal). Scale bars = 200 μm. **c**, Schematic representation of human colonic double-layered mucus. **d**, Representative fluorescent micrographs of Fmoc-F_F_:PFD structures in the upper (top) and lower (bottom) material layers. A * indicates coacervate droplets. Scale bars = 50 μm. **e**, Representative scanning electron microscopy images of the Fmoc-F_F_ fibrillar gel upper layer, at varying magnification. Scale bars (from left to right) = 200, 20, 2 and 0.2 μm.

While screening a surfactant library to prepare fluorous emulsions, we discovered the ability of the non-natural amino acid, Fmoc-pentafluoro-L-phenylalanine (Fmoc-F_F_), to form viscous, mucus-like, gels at liquid-liquid phase separated interfaces (Fig. 1a). The material is generated by pipetting a solution of perfluorodecalin (PFD) containing Fmoc-F_F_ (20 mmol/L) into saline, leading to the spontaneous supramolecular organization of the amino acid at the fluorous-water interface. This rapid and irreversible step produces a dense coacervate gel, from which a fibrillar network evolves into the aqueous fraction when incubated at 37°C (Fig. 1b). This double layered structure mimics the typical morphology of colonic mucus, which is characterized by a dense inner layer that adheres to epithelial cells and a diffuse fibrillar outer layer that harbors commensal bacteria (Fig. 1c). To further visualize the bilayer composition of our synthetic mucus, the lipophilic dye nile red was dissolved in the PFD solvent before gel fabrication. Thioflavin T (ThT), a dye that displays enhanced fluorescence upon binding to amyloid structures,^15^ was added to the aqueous fraction to label Fmoc-F_F_ assembled fibrils. Representative fluorescent micrographs shown in Fig. 1d demonstrate that the bottom layer of the material is a tightly packed network of PFD droplets, from which Fmoc-F_F_ assembly initiates at the fluorous-water interface. Co-localization of nile red and ThT signals suggests these interfacial structures are amorphous hydrophobic oligomers and/or protofibrils, representing the nascent stages of Fmoc-F_F_ assembly. Conversely, the upper layer is a diffuse collection of extended fibers characterized only by ThT fluorescence, with a marked absence of nile red (Fig. 1d, top). This suggests these are mature, organized fibrils that have evolved from rearrangment of the disorganized structures templated in the lower coacervate layer. Parallel electron microscopy, shown in Fig. 1e, further demonstrates that the less dense upper layer is an interpenetrating system of Fmoc-F_F_ self-assembled fibrils. The cohesivity of this layer is imparted via non-covalent entanglement of fibers, creating a porous mesh-like architecture.

**Figure 2:**
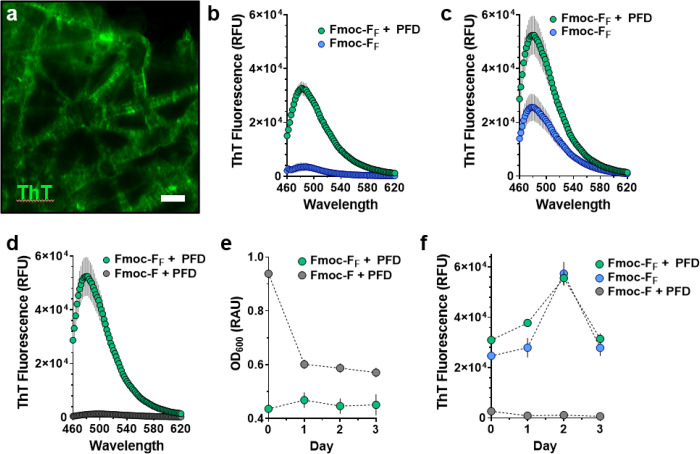
Mechanistic investigation of Fmoc-F_F_ assembly. **a**, Representative fluorescent micrograph of ThT-stained Fmoc-F_F_ fibrillar bundles. Scale bar = 40 μm. **b**,**c**, Average fluorescence spectra of ThT dye in the presence of Fmoc-F_F_ pre-dissolved in PFD before creating liquid-liquid phases in saline (green circles) or direct dissolution of Fmoc-F_F_ in saline alone (blue circles) at 1 (**b**) and 2 (**c**) days of incubation. RFU = Relative Fluorescence Units. **d**, Average fluorescence spectra of ThT dye in the presence of Fmoc-F_F_ (green circles) or non-fluorinated Fmoc-F (grey circles) amino acids assembled at PFD-saline interfaces after 2 days of incubation. **e**,**f**, Time-dependent optical density (**e**, OD_600_) and ThT fluorescence (**f**) profile of amino acid assemblies prepared under the indicated conditions. Data shown in panels b – f represents average ± s.e.m. of n = 3 replicates.

Next, we investigated the nature and rate of molecular organization of Fmoc-F_F_ assemblies using the ThT dye. The multilamellar appearance of ThT-stained fibers suggests they are composed of β-sheet like plates that organize through n→π* stacking of Fmoc-F_F_ residues (Fig. 2a).^16, 17^ This organization is strongly induced by the presence of the phase-separated PFD droplets, as demonstrated by the attenuated ability of Fmoc-F_F_ to form fibers when PFD is absent in the saline solution (Fig. 2b). Here, the PFD-aqueous interface serves to rapidly template the assembly of Fmoc-F_F_ monomers into a stable gel, from which fibers continue to grow and evolve over several days (Fig. 2c).

To further investigate this assertion, and specifically isolate fluorine-fluorine driven effects, we evaluated the assembly of non-fluorinated Fmoc-L-phenylalanine (Fmoc-F) under similar conditions (Fig. 2d). Results show this fluorine-deficient analogue is not capable of forming viscous fibrillar gels and instead generates colloidal emulsions, as indicated by the combination of high optical density (Fig. 2e) and low ThT fluorescence (Fig. 2f). Prior studies from our group suggest these divergent assembly pathways result from the propensity for perfluorocarbon-water systems to preferentially direct J-aggregate formation of Fmoc-F_F_, and not the non-fluorinated Fmoc-F analogue.^17^ This leads to Fmoc-F_F_ being uniquely organized into anti-parallel arrangements, where fluorenyl moieties form alternate β-sheets to create π-stacked pairs with interleaved fluorinated phenyl rings. Propagation of these stacked assemblies likely yields the observed fibrils.^17^ Given the unique hierarchical organization of Fmoc-F_F_, induced by the presence of the perfluorinated phenyl ring and perfluorocarbon droplet interface, we hereafter refer to the assembled material as fluorine-assisted mucus surrogate, or FAMS.

**Figure 3:**
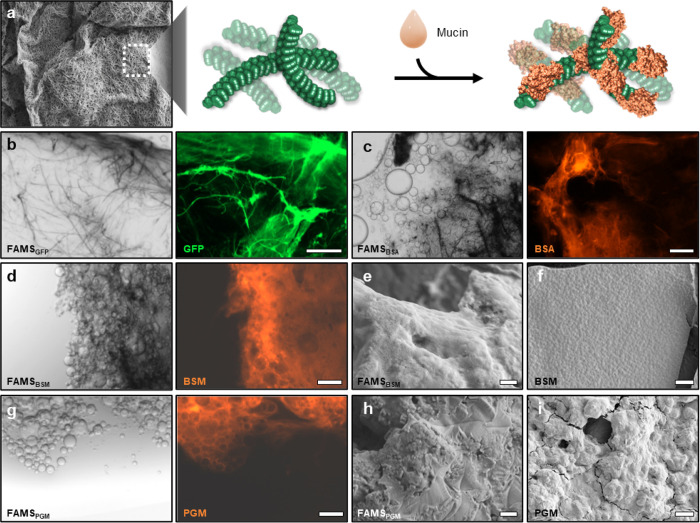
Protein-coating of FAMS gels. **a**, Schematic representation of non-covalent mucin coating of the FAMS fibrillar surface (SEM micrograph reused from Figure 1e). **b**, **c**, Representative optical (*left*) and fluorescent (*right*) micrographs of GFP (**b**) and Cy5-labeled BSA (**c**) coated FAMS. Scale bars = 200 μm. **d**-**i**, Representative optical (*left*) and fluorescent (*right*) micrographs of Cy5-labeled bovine submaxillary mucin (**d**, BSM) and porcine gastric mucin (**g**, PGM) coated FAMS. Associated scanning electron microscopy images of FAMS surface after BSM (**e**) or PGM (**h**) coating. SEM images of native BSM (**f**) and PGM (**i**) reconstituted mucin gels shown for comparison. Scale bars in panels d and g = 200 μm. Scale bars in remaining panels = 1 μm.

To prepare the surface of FAMS for bacterial attachment we next coated the fibrils with mucin proteins (Fig. 3a). Here, simple addition of a protein solution to pre-formed FAMS led to rapid and robust fibril adsorption, producing a protein surface coating that was stable to multiple washes with media. Optimization of the coating procedure was done using two model fluorescent proteins, GFP and Cy5-BSA. Fluorescence microscopy demonstrated these proteins non-covalently decorate the materials and are retained after multiple washing (Fig. 3b, c). Similar studies were then performed using Cy5-labeled bovine submaxillary mucin (BSM) and porcine gastric mucin (PGM). As expected, addition of BSM lead to uniform coating of the material network (Fig. 3d), with additional SEM imaging demonstrating a relatively smooth surface topography to BSM-coated FAMS (FAMS_BSM_, Fig. 3e). This resembled the topology of reconstituted mucus prepared from the BSM protein stock (Fig. 3f), suggesting the mucin assembles into cohesive sheets that envelop the FAMS fibrillar network. Coating with PGM was similarly successful (Fig. 3g), although the surface morphology was more irregular (Fig. 3h) due to the uneven assembly of PGM itself (Fig. 3i). SEM imaging confirmed the morphology of BSM and PGM coatings was consistent across multiple length scales (Supporting Figs. 1 and 2). Taken together, our data indicates that addition of mucins does not disrupt the integrity of the FAMS super-structure, producing a mucin-rich double layer material that approximates the structural and biochemical characteristics of colonic mucus.

## Mechanical Analysis of Mucin-Functionalized FAMS

A key checkpoint for mimicry of GI colonic mucus is material viscoelasticity, as the ability of native mucus to flow over long loading periods is necessary for the movement of solids during peristalsis. Mucus viscosity also has important implications in shaping microbial behavior and contributes to disease. For example, *H. pylori* can alter environmental pH to reduce the viscoelasticity of gastric mucus, thereby compromising the integrity of its barrier function.^18^ Similarly, several intestinal microbial pathogens secret proteases that degrade MUC2 to modulate mucus viscosity during pathogenesis,^19^ leading to pro-inflammatory contact between gut flora and immune cells.^20^

**Figure 4:**
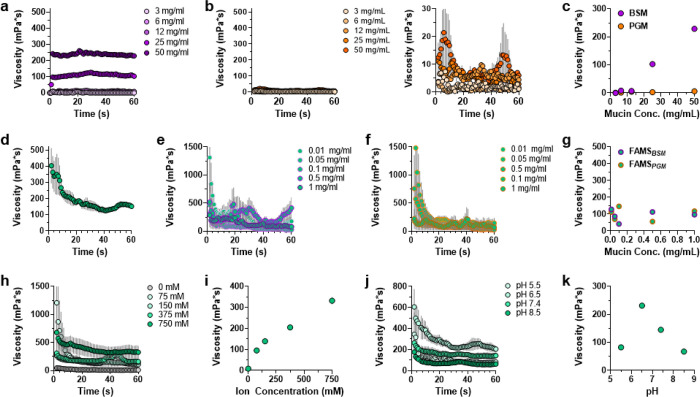
Rheological characterization of FAMS. **a**, **b**, Time- and concentration-dependent dynamic viscosity curves for BSM (**a**) or PGM (**b**) reconstituted gels. Panel b, left, shows viscosity profile of PGM at a matched y-axis scale to panel a for comparison. Panel b, right, shows a reduced y-axis scale to better visualize curves. **c**, Concentration-dependent equilibrium viscosity for BSM (purple) or PGM (orange) reconstituted gels. **d**, Time-dependent dynamic viscosity profile of FAMS gels. **e**, **f**, Time-dependent dynamic viscosity of FAMS gels coated with varying concentrations of BSM (**e**) or PGM (**f**). **g**, Concentration-dependent equilibrium viscosity for FAMS gels coated with BSM (FAMS_BSM_) or PGM (FAMS_PGM_). **h**, **j**, Time-dependent dynamic viscosity of uncoated FAMS gels in phosphate buffered saline at varying salt concentration (**h**) or pH (**j**). **i**, **k**, Concentration-dependent equilibrium viscosity for uncoated FAMS gels as a function of ion concentration (**i**) or pH (**k**). Data shown represents average ± s.e.m. of n = 3 replicates.

Recent rheologic studies show healthy human colonic mucus is composed of 1.3–1.9 wt% (13–19 mg/mL) mucin solids, yielding a dynamic viscosity of 150–250 mPa*s.^21^ Using this benchmark, our first mechanical characterization step was to assess the baseline viscosity for the mucins themselves when reconstituted in saline (Fig. 4a–c). Rheological dynamic time sweep measurements of reconstituted BSM showed a dynamic viscosity of 1–228 mPa*s as mucin concentration was increased from 3–50 mg/mL, resembling the rheological performance of native human colonic mucus.^21^ PGM was less viscous, reaching an average dynamic viscosity of 6 mPa*s at the highest tested concentration. We next tested the viscosity of native FAMS without mucin coating. Results in Fig. 4d demonstrate a prolonged stress relaxation response of the viscoelastic material over the first 40 seconds of loading, reaching a plateau dynamic viscosity of approximately 150 mPa*s. This behavior is similar to the long stress-relaxation response reported for porcine gastric mucus gels.^22^ Mucus viscoelasticity results from reversible, non-covalent interactions between mucin components, enabling solid-like responses over short loading periods and flow behavior on longer time scales. Interestingly, while FAMS was able to replicate the viscoelastic nature of native mucus, we did not observe this same flow behavior for reconstituted BSM and PGM (Figs. 4a,b). This is likely due to the compositional simplicity of these solutions, which do not replicate the varied biomolecular constituents and gradient structural morphology of native gut mucus. With these benchmarks established, we next tested the mechanical performance of FAMS coated with BSM (Fig. 4e) and PGM (Fig. 4f) mixtures. Rheological measurements demonstrate that, although the relaxation behavior of native FAMS is retained, the initial dynamic viscosity magnitude is greater when the gels are coated with mucins than without. Still, mucin-coated FAMS were found to maintain a ~ 100 mPa*s viscosity plateau regardless of the loaded mucin concentration (Fig. 4g).

Given the sensitivity of GI mucus to changes in environmental ionic strength and pH, we next tested these parameters on the viscosity of FAMS. Increasing the total salt concentration of the phosphate buffered saline environment from 0–750 mM led to a corresponding increase in FAMS viscosity (Fig. 4h, i). This is converse to native mucus, where increasing ion strength generally correlates to reduced viscosity due to polyelectrolyte charge-shielding.^23^ In our case, screening of the Fmoc-F_F_ anionic charge by salt may alter its solubility and shift its kinetic equilibrium in favor of fibrillar assembly, thereby producing a more cohesive and viscous FAMS gel. Changing solution pH also led to variable FAMS viscoelastic responses (Fig. 4k). Here, average dynamic viscosity increased from 83 mPa*s at pH 5.5 to 231 mPa*s at pH 6.5, before declining as the solution became more basic. This bears resemblance to native mucus, which exhibits decreasing viscosity as environmental pH transitions from neutral to weakly alkaline.^24^ The ability of FAMS to mechanically respond to its environment is most likely regulated by the protonation state of Fmoc-F_F_’s carboxylic acid. This is supported by studies on various Fmoc-Phe derivatives demonstrating that changes in ionic strength and pH modulate Coulombic repulsion between anionic charged amino acids to alter their assembly propensities.^16, 25^ In sum, our results show that the viscoelastic behavior of FAMS can be modulated by environmental conditions to create mucus analogues with customizable rheologic properties that match native colonic mucus.

## Development of Synthetic Microbiome Organoids

**Figure 5:**
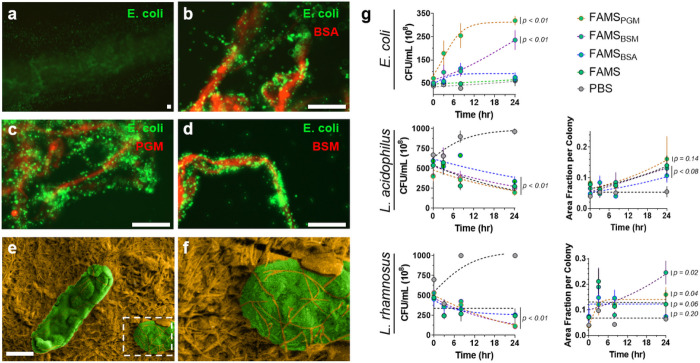
Commensal colonization of FAMS gels. **a-d**, Representative fluorescent confocal micrographs of GFP-expressing *E. coli* (green) seeded onto uncoated FAMS (**a**), or materials coated with Cy5-labeled (red) BSA (**b**), PGM (**c**) or BSM (**d**). Scale bars in panel a – d = 500 μm. **e**, False colored scanning electron micrograph of *E. coli* (green) colonizing FAMS (orange). Scale bar = 500 nm. **f**, Magnified region of interest from panel e (dashed white box) demonstrating fibrillar entanglement of colonizing *E. coli* cells. **g**, Colony formation assays for the indicated gastrointestinal microbe inoculated into blank phosphate buffered saline (PBS, grey) or the indicated FAMS formulation. Plots for *L. acidophilus* and *L. rhamnosus* show time-dependent change in CFU/mL (*left*) and relative colony size (*right*). Data shown represents average ± s.e.m. of n = 3 replicates. p values for each formulation are shown next to the respective data and evaluated at the final time point (24 hour) relative to PBS control.

Microbial integration into FAMS was initially investigated using a stably expressing GFP-*E. coli* strain to aid visualization. Although *E. coli* was able to bind to the surface of uncoated FAMS, its attachment was relatively poor as indicated by low cellular fluorescence (Fig. 5a). Conversely, mucin coatings led to significant *E. coli* colonization on, and within, the fibrillar scaffold, forming dense microbial communities (Fig. 5b–d, Supporting Fig. 3). Scanning electron microscopy confirmed that the microbes don’t simply reside at the surface of the material, but integrate within the fibrillar mesh (Fig. 5e,f, Supporting Fig. 4).

Next, growth studies evaluated the proliferation of *E. coli* seeded onto the FAMS materials after a 24 hour incubation (Fig. 5g). These assays were performed in sterile PBS to minimize environmental nutrients, and thereby allow us to isolate the effects of FAMS coatings on *E. coli* growth trends. Results show the mucin-coated FAMS formulations (e.g., FAMS_PGM_, FAMS_BSM_) supported logarithmic growth of colonizing *E. coli*, with PGM coatings leading to more rapid microbial growth than BSM. Control FAMS prepared with BSA, which serves as a non-mucin protein control (FAMS_BSA_), and the naked material alone (FAMS) showed a significantly blunted growth profile. This suggests that *E. coli* can utilize the loaded mucins as a nutrient source to support robust colonization and growth within the coated FAMS materials.

While these results are encouraging, *E. coli* is considered a gastrointestinal pathobiont and so we next tested the canonical probiotic commensals *L. acidophilus* and *L. rhamnosus* (Fig. 5g). Visual microscopic inspection of inoculated FAMS gels showed these anaerobic strains more deeply integrated within the synthetic mucus bulk relative to *E. coli*, likely to minimize their exposure to oxygen in solution. Although the media used for these studies contains an L-cysteine reducing agent, the solution is not completely anoxic. As a result, we found that *L. acidophilus* and *L. rhamnosus* were difficult to extract from the entangled fibers during plating assays, leading to an apparent decline in the measured CFU/mL over 24 hours (Fig. 5g, lower left plots). However, both strains showed a parallel increase in the size of colonies formed on the plated media (Fig. 5g, lower right plots), suggesting cohesion of plated bacteria by FAMS fibers. This indicates that the reduction in plated CFU/mL for these strains may be due, in part, to association of the bacteria with the FAMS network, thereby inhibiting its transfer to the agar surface and creating larger seed colonies. In sum, our results suggest that gastrointestinal pathobionts and commensals can successfully integrate into FAMS and colonize the synthetic mucus network to create microbial communities.

**Figure 6:**
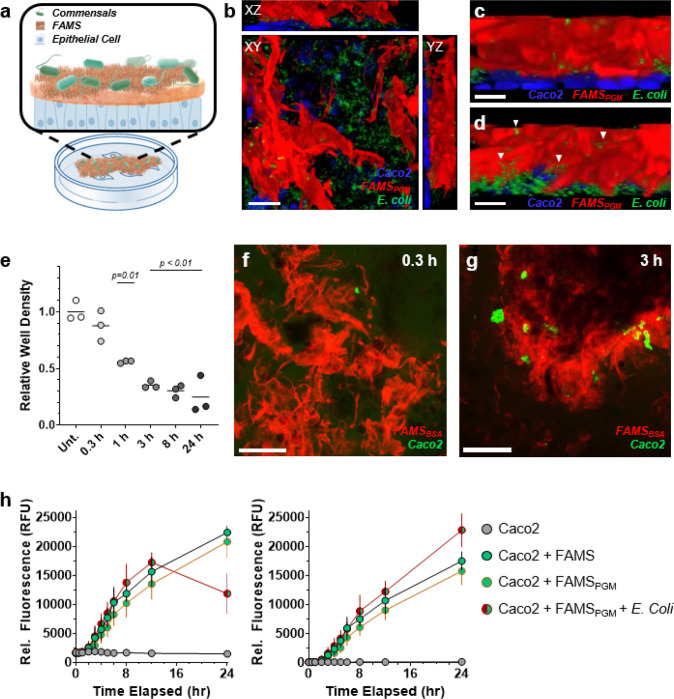
Synthetic microbiome organoids. **a**, Conceptual schematic of artificial colonic mucosa in a high throughput well plate format. **b**, Orthographic fluorescent confocal micrographs of synthetic microbiome containing human colorectal Caco2 cells (blue), FAMS_PGM_ (red) and GFP-expressing *E. coli* (green). Scale bar = 20 μm. **c**, **d**, Magnified z-stack images of artificial mucosae highlighting FAMS integration with Caco2 epithelial monolayer (**c**) and colonization by inoculated *E. coli* (**d**, white triangles). Scale bars in panels c and d = 5 μm. **e**, Relative Caco2 cell density remaining on transwell surface after varying incubation times with FAMS_PGM_. Data shown represents average ± s.e.m. of n = 3 replicates. p values for all time points are ≤0.01 relative to the untreated control (Unt.). **f**, **g**, Confocal micrographs of FAMS_PGM_ removed from the surface of Caco2 cell monolayers after 0.3 (**f**) and 3 (**g**) hours of incubation. Viable Caco2 cells are stained with calcein-AM (green). Scale bars = 200 μm. **h**, Time-dependent apical-to-basolateral diffusion of 4kDa FITC-dextran (left) and 70kDa rhodamine-dextran (right) across transwell-seeded Caco2 cell monolayers without (grey circle) or with integrated FAMS (green circle), FAMS_PGM_ (green circle/orange border) or FAMS_PGM_ and *E. coli* mixture (green/red circle). Data shown represents average ± s.e.m. of n = 3 replicates. All confocal micrographs shown are a representative image from replicate analyses.

Encouraged by these results, we set out to develop a simple, rapid, and low-cost fabrication protocol to generate multilayer microbiome organoids suitable for high throughput screening applications (Fig. 6a). We began by creating a colorectal epithelial layer using human Caco2 cells cultured for ≥ 19 days on transwell inserts. Appearance of tight junctions between cells in the monolayer, as indicated by Occludin staining (Supporting Fig. 5), confirmed the formation of an organized epithelial interface. We then added the FAMS_PGM_ mucus analogue and inoculated with *E. coli* to create the final microbiome model. Orthographic microscopy images shown in Fig. 6b demonstrate the three-dimensional layering of each component in the synthetic microbiome. Like native colonic mucosae, FAMS was adhered to the surface of colorectal cells (Fig. 6c) and was permeated throughout the z-plane by colonizing microbes (Fig. 6d). Surprisingly, adherence of the Caco2 cell monolayer to FAMS was rapid, leading to sufficient transfer/migration of the cells with/into the fibrillar assembles to remove them from the transwell surface (Fig. 6e). Additional imaging studies showed this adhesion was significant after ≥ 1 hour of incubation, and that FAMS-adhered Caco2 cells remained viable and metabolically active (Fig. 6f,g). Although we cannot conclusively rule out some level of Caco2 cell death in these models, our data strongly supports the assertion that Caco2-FAMS-microbe mixtures form a tightly integrated system that reproduces many of the practical structural and morphologic features of native colonic mucus.

The relative ease with which these FAMS-enabled model colonic microbiomes can be constructed highlights their potential for screening applications. As an exemplary demonstration, we tested the permeation of low (4kDa) and high (70kDa) molecular weight dextran dyes through the FAMS generated model mucosae (Fig. 6i). Permeability assays showed that both the 4kDa and 70kDa markers were unable to diffuse across control, unmodified, Caco2 monolayers over the 24-hour incubation period (see grey circles in Fig. 6i). Conversely, Caco2 monolayers layered with FAMS, either uncoated (FAMS) or functionalized with PGM (FAMS_PGM_), showed an increase in cumulative dextran basolateral diffusion at the 2-hour incubation time point, which then generally increased monotonically with time. The notable exception was for the complete Caco2-FAMS_PGM_-*E. coli* mixture, which showed a decline in 4kDa basolateral fluorescence between the 12- and 24-hour measurement time points (see left plot in Fig. 6i). We ascribe this reduction to a metabolism of the dextran dye by *E. coli* at these longer time intervals. Several gastrointestinal bacteria have been reported to metabolize dextran as a nutrient source;^26, 27^ although we were unable to find a specific reference for the *E. coli* strain used in these assays (101–1). The 70kDa dextran did not show the same decline in fluorescence during the 12–24 hour interval, suggesting it’s higher molecular weight inhibited enzymatic processing.

*In vivo* pharmacokinetic studies report that 4kDa dextran permeates the gut and enters systemic circulation as early as 15 minutes after oral gavage, achieving maximum plasma concentration at 1–4 hours; dependent on mouse strain.^28^ The same study described slowed gastrointestinal transit kinetics for 70kDa dextrans compared to 4kDa markers, with serum bioavailability presumably similarly delayed. Taken in context with our data (Fig. 6i), FAMS-generated microbiome organoids appear significantly better at mimicking the *in vivo* pharmacokinetics of dextran than unfunctionalized Caco2 monolayers, which currently are considered the gold standard for *ex vivo* drug permeability assays.^29^ This is most likely due to mechano-chemical cross-talk between FAMS, *E. Coli* and Caco2 cells, leading to a more permissive mucosal interface that may better replicate the *in vivo* environment.

## Outlook

Here, we exploit privileged fluorine-fluorine interactions to template the assembly of a fluorinated amino acid at liquid-liquid phase separated interfaces. The resultant supramolecular matter (fluorine-assisted mucus surrogate, FAMS) is a viscous gel that mimics the double layer architecture of native human colonic mucus. Rheological studies show FAMS resembles the viscoelastic properties of colonic mucus, and can be tuned in its mechanical performance via controlling environmental ionic strength and pH. Added mucin proteins rapidly adhere to the surface of the fibrillar network to simulate the proteinaceous profile of mucus without disrupting the viscoelastic properties of FAMS. Together, this generates a colonic mucus surrogate that can be inoculated with gastrointestinal pathobionts or commensals, and added to human colorectal epithelium, to generate a multicellular synthetic microbiome. We envision these materials will provide a simple, robust, and tractable tool to study commensal biology and microbe-mucus-host interactions *ex vivo*. The addition of leukocytes may expand this platform to advance our understanding of microbe-immune cell interactions in the gut. Since FAMS utilizes inexpensive and commercially available building blocks, are generated using simple protocols that do not require specialized equipment, and can be integrated with established epithelial models, these materials may have utility in high throughput screening campaigns. As an exemplary application, we show that FAMS can be readily incorporated into standard Caco2-based permeability assays to enhance the prediction of *in vivo* drug adsorption. Because these FAMS-enabled models can be developed without significant cost or training they represent a complementary alternative to microfluidic and stem cell-based organoid approaches that are currently limited in their broad utility due to complexity and cost.

## METHODS

### Materials:

Fmoc-pentaflurophenylalanine and thiazolyl blue tetrazolium bromide (MTT) were purchased from Chem Impex. Perfluorodecalin (PFD) and Thioflavin T (ThT) were purchased from Oakwood Chemical. Phosphate buffered saline (PBS, 1x) without calcium and magnesium, Dulbecco’s Modified Eagle’s Medium (DMEM) with 4.5 g/L glucose, L-glutamine, and sodium pyruvate, DMEM with 4.5 g/L glucose and sodium pyruvate, without L-glutamine and phenol red, Falcon 75 cm^2^ cell culture flasks, Falcon 1.0 μm pore PET transwell membranes, 96-well tissue culture plates, and Trypan blue 0.4% w/v were purchased from Corning. PBS 10x without calcium and magnesium, bovine serum albumin, LB Miller Broth (granulated), Mueller Hinton Broth 2, bacteriological agar, 6– 12- and 24- well tissue culture plates, Nunc Lab-Tek II 4-well chambered coverglass, Prolong Diamond Mountant with DAPI, Gibco 100x Minimum Essential Medium Non-Essential Amino Acids (MEM NEAA), Gibco Trypsin-EDTA 0.25%, Dimethylsulfoxide (DMSO), Slide-a-lyzer 3.5 kDa molecular weight cutoff (MWCO) dialysis cassettes, and adhesive sheets were purchased from Thermo Fisher Scientific. Bovine submaxillary mucin (Type I-S, BSM), mucin from porcine stomach (Type II, PGM), Penicillin-Streptomycin, L-cysteine HCl, Fluorescein isothiocyanate (FITC) Dextran (~ 3,000–5,000 Da), Rhodamine B isothiocyanate Dextran (70,000 Da) were purchased from Sigma Aldrich. Fetal Bovine Serum (FBS) was purchased from VWR. Sulfo-Cy5 NHS ester was purchased from Lumiprobe. Calcein AM was purchased from Cayman Chemical. Rabbit anti-occludin primary antibody and AlexaFluor 488 Goat Anti-Rabbit secondary antibody were purchased from Abcam. Paraformaldehyde (PFA, 4%) was purchased from Santa Cruz Biotechnology. Ampicillin was purchased from Dot Scientific. Difco Lactobacillus MRS broth and Difco Lactobacillus MRS agar were purchased from BD Biosciences.

Caco2 colorectal adenocarcinoma cells, *Lactobacillus acidophilus* (4356), and *Lactobacillus rhamnosus* (9595) were purchased from ATCC. *Escherichia coli* 101–1 was a gift from Dr. Kenneth Keiler at The Pennsylvania State University. Rosetta 2 (DE3) *Escherichia coli* expressing green fluorescent protein (GFP *E. coli*) and recombinantly expressed GFP were gifts from Dr. Joel Schneider at the National Cancer Institute.

### FAMS Assembly

Fmoc-F_F_ was dissolved in room-temperature PFD at 20 mM by vortex mixing, then ultrasonication for 10 minutes. Fmoc-F_F_ in PFD solution was added to PBS 1x at 1:10 v:v. This mixture was then emulsified by vortex mixing at 3000 rpm for 1 minute. Sealed samples were incubated at 37° C for 0–72 hours before use. Samples for biological and rheological studies were incubated for 48 hours before use.

### Rheology

Viscoelastic properties of FAMS were measured on a ThermoHaake RotoVisco 1 with cone-and-plate format (L01019C60/1 Ti). BSA, PGM, and BSM were each dissolved to their desired concentration in PBS 1x. Equal volume (2 mL) of each sample was pipetted onto the center of the plate. To avoid disruption of FAMS structure, 2 mL volume samples were constructed in sealed, inverted 24-well microwell plates. The seal was removed to deposit the FAMS sample directly to the center of the plate. Viscosity was measured at constant deformation of 10 Hz for 60 seconds, capturing data at initiation and once per second thereafter. Each condition was repeated in triplicate (n = 3). Dynamic viscosity was calculated by dividing the measured viscosity at each timepoint by the recorded deformation rate.

### Fluorescent Spectroscopy and Imaging

Fibrillar assembly was tracked by monitoring ThT binding over several days via fluorescence spectroscopy. Stock ThT solution was created by dissolving powdered ThT in PBS 1x to 2.5 mM. Homogenous ThT solution was added to freshly mixed FAMS to final concentration of 0.05 mM, and samples were incubated at 37° C for 72 hours. ThT activity was tracked by measuring fluorescence across each sample with a Biotek Cytation 3 Imaging Plate Reader. Samples were excited at 430 nm and emission was recorded at 490 nm. Images of fibers with ThT were captured with a Cytation 3 in Brightfield and GFP (excited at 469 nm, detected at 597 nm) channels.

Imaging of protein adsorption to FAMS was performed by first fluorescently labeling BSM, PGM, and BSA with Sulfo-Cy5 NHS Ester. Each protein was dissolved to 5 mg/mL in 0.1 M NaHCO_3_ buffer, before addition of Sulfo-Cy5 NHS Ester in 8 molar excess. Protein solution was vortex mixed with Sulfo-Cy5 NHS solution and kept on ice overnight. Unbound Sulfo-Cy5 NHS Ester was removed by dialysis in 3.5 kDa MWCO cassettes against pure DI water for 24 hours. Purified Cy5-tagged protein solutions were frozen at −80°C overnight, lyophilized to obtain dry powder, and stored at 4°C until use.

Cy5-tagged proteins were dissolved at 1 mg/mL in PBS 1x by vortex mixing and 1 hour of shaking at 37° C. Protein solution was added to complete FAMS to achieve final concentration of 0.02 μg/mL protein in FAMS, then gently mixed. Samples were extracted by pipetting and placed into clean microwell plates for imaging. Fluorescent and brightfield images were captured using a Biotek Cytation 3 in the Texas Red channel (excitation at 586 nm, detection at 647 nm).

### Bacterial Growth and FAMS Inoculation

GFP *E. coli* was cultured overnight in LB Miller Broth with 50 μg/mL ampicillin, shaking at 37°C. *E. coli* 101–1 was cultured overnight in Mueller Hinton Broth 2, shaking at 37°C. *L. rhamnosus and L. acidophilus* were cultured overnight in Difco Lactobacillus MRS Broth in stationary capped tubes at 37° C. Each liquid culture was centrifuged at 6000 × g for 10 minutes to pellet and resuspended in PBS 1x before use in assays. Growth was assessed by optical density (OD) measurements at 600 nm in polystyrene cuvettes using a ThermoScientific Genesys 150 UV-Visible Spectrophotometer. Solution density was normalized to OD = 1.00 in PBS 1x for each bacterial sample to control volume variability in all experiments.

Microbes were cultured in FAMS and supplementary proteins to track survivability. Treatment wells of FAMS were produced in sealed 24-well plates as described above, 1 mL in volume. Wells receiving additional proteins were inoculated with 10 mg/mL protein solution (PGM, BSM, BSA) to achieve a final concentration of 0.5 mg/mL. Density-normalized bacterial solution, prepared as above, was used to inoculate each treatment well with final theoretical OD = 0.01. For *E. coli*, no additional supplements were added to the treatment wells. For both *Lactobacillus* species, treatment wells were supplemented with 100 μL Lactobacillus MRS broth and 5 μM L-cysteine to partially reduce the growth environment. Cultures remained in treatment solution for 0–24 hours before sampling. Wells were sampled, diluted, and plated onto 50 mm agar plates containing supplement appropriate to the species. For *E. coli* 101–1, plates contained LB Miller broth mixed with bacteriological agar. For both *Lactobacillus* species, plates contained Lactobacillus MRS agar. Dilutions were optimized for each species to facilitate colony forming unit (CFU) counting assays. After 24–48 hours of incubation, each plate was photographed. ImageJ was used to analyze the images to obtain a metric of CFU/mL for each sample.

### Electron microscopy

Scanning electron microscopy (SEM) was accomplished by drying samples (20 μL) of FAMS, protein-coated FAMS, and *E. coli* – loaded FAMS on 12.7 mm Aluminum specimen mounts (Ted Pella, Inc., 6 mm Pin, Zeiss). Samples were gold-palladium coated using a Bal-tec SCD-050 sputter coater. Micrographs were obtained using a Zeiss VP-FESEM with electron high tension of 3.5 kV and working distance 5.3 mm. Facilities used for SEM were courtesy of the Huck Institutes of Life Sciences Microscopy Facility at The Pennsylvania State University. Images were false colored in Adobe Photoshop where indicated.

### Confocal imaging of synthetic microbiome organoids

Models of synthetic microbiome organoids were made using FAMS, colorectal cancer cells, fluorescently-tagged mucins, and fluorescent bacteria. Colorectal cancer cells (Caco2) were seeded onto 4-well chambered cover glass at 1.5×10^5^ cells/cm^2^. Culture media was DMEM with 4 mM L-glutamine, 10% FBS, Penicillin-Streptomycin (100 units and 0.1 mg/mL, respectively), and 1x MEM NEAA. Cells were cultured to 80% confluence, changing media every 4 days. Cell monolayer was fixed with 4% PFA for 15 minutes, washed twice with PBS 1x, then nuclei were stained with Hoechst 33342 (5 μg/mL). After 20 minutes, cells were washed twice with PBS 1x. Pre-made FAMS was added to the chamber wells by pipetting. Cy5-PGM was added in solution to achieve final concentration of 0.5 mg/mL in the chamber wells. Density-normalized solution of GFP *E. coli* was added to chamber wells to a theoretical OD = 0.01. Synthetic microbiome organoids were imaged using a Zeiss LSM 880 Airyscan Fast at the Huck Institutes of Life Sciences Microscopy Facility at The Pennsylvania State University. Images were captured in three channels using Zeiss Zen Black microscopy software: Hoechst 33342 (excited at 405 nm, detected between 410 and 483 nm), EGFP (excited at 488 nm, detected between 493 and 597 nm), and Cy5 (excited at 633 nm, detected between 638 and 759 nm).

### Transmembrane diffusion

Colorectal cancer cells (Caco2) were seeded onto 1.0 μm pore PET transwell membranes in 6-well plates at 1.5×10^5^ cells/cm^2^. Cells were cultured 19–23 days until immunostaining for occludin via application of rabbit anti-occludin primary antibody for 30 minutes at 1 μg/mL, followed by Alexa Fluor^®^ 488 Goat Anti-Rabbit IgG H&L for 30 minutes at 7.5 μg/mL. Transwell membranes were cut from the membrane chamber using a surgical blade. The membranes were fixed to glass slides with ProLong Diamond Mountant with DAPI and covered with #1 glass covers. Slides were imaged by confocal microscopy with a Zeiss LSM 880, with excitation at 488 nm and detection at 495–630 nm. After satisfactory tight junction formation had been achieved, media was removed and 2 mL FAMS material including PGM (0.5 mg/mL) and 101–1 *E. coli* (theoretical OD = 0.01) was added in the apical chamber. The basolateral chamber was filled with 3 mL phenol red-free DMEM as formulated above. FITC-labelled (~ 3,000–5,000 g/mol) and Rhodamine B-labelled (70,000 g/mol) dextrans were added to achieve 5 μM concentration of each in the apical chamber. Sample volumes were collected from the basolateral chambers of each transwell assembly over 24 hours. Plates were kept at 37° C, 5% CO2 between samplings. Plates containing *E. coli* were kept in an independent incubator at similar conditions. Diffusion was measured by fluorescence of the basolateral media samples, in both FITC (excitation 488 nm, detection 525 nm) and Rhodamine B (excitation 586 nm, 647 nm).

### Cell detachment

Caco2 monolayers were cultured on transwell membranes as described above. After incubation with FAMS at varying timepoints, transwells were washed with three times with PBS 1x. MTT solution was added to each well to achieve final concentration of 0.5 mg/mL, then incubated at 37° C for 3 hours. MTT solution was removed and replaced with 100% DMSO, then MTT crystals were dissolved by shaking for 15 minutes at room temperature. Absorbance was measured across the area of each well at 570 nm using a Biotek Cytation 3.

To assess cell attachment/migration onto/into FAMS, Caco2 cells were cultured similarly in 12-well plates. At 80% confluence, cells were treated with FAMS and Cy5-BSA (0.5 mg/mL), then incubated at 37° C, varying duration. Wells were then treated with Calcein AM (5 μM final conc.) for 30 minutes. Samples for imaging were extracted by pipetting the supernatant FAMS from each well into a clean 12-well plate. Samples were imaged by confocal microscopy with a Zeiss LSM 880, exciting at 488 nm for Calcein AM (detected 493–616 nm) and 633 nm for Cy5-BSA (detected 638–759 nm).

### Statistical analyses

Experiments are represented as the mean of independent replicates, with standard error of the mean (s.e.m.) where indicated in the figure caption. Data was analyzed using GraphPad Prism 9 software. P values, where shown, are indicators of significance obtained from unpaired Student’s t-tests using equal variance.

## Data Availability

Data collected for this study is found in the text or in the Supplementary Information. Raw data is available upon reasonable request to the corresponding author.
